# Trends in Azole Resistance in *Aspergillus fumigatus*, the Netherlands, 1994–2016

**DOI:** 10.3201/eid2501.171925

**Published:** 2019-01

**Authors:** Jochem B. Buil, Eveline Snelders, Laura Bedin Denardi, Willem J.G. Melchers, Paul E. Verweij

**Affiliations:** Radboud University Medical Center, Nijmegen, The Netherlands (J.B. Buil, W.J.G. Melchers, P.E. Verweij);; Center of Expertise in Mycology Radboudumc/CWZ, Nijmegen (J.B. Buil, W.J.G. Melchers, P.E. Verweij);; Wageningen University and Research, Wageningen, the Netherlands (E. Snelders);; Federal University of Santa Maria, UFSM, Santa Maria, Brazil (L.B. Denardi)

**Keywords:** azole resistance, A*spergillus fumigatus*, aspergillosis, epidemiology, antimicrobial resistance, fungi, the Netherlands

## Abstract

We investigated azole resistance in *Aspergillus fumigatus* in a tertiary reference hospital in the Netherlands during 1994–2016. The 5-year patient-adjusted proportion of resistance increased from 0.79% for 1996–2001 to 4.25% for 2002–2006, 7.17% for 2007–2011, and 7.04% for 2012–2016. However, we observed substantial variation between years.

Azole resistance is increasingly reported in *Aspergillus fumigatus* and is now found all around the world (*1*). Most studies have investigated the presence of resistance in environmental or clinical samples over a limited time, but longitudinal resistance studies are lacking. It is important to determine if azole resistance frequency shows increasing trends over time and if the distribution of resistance mutations changes over time. We have previously reported the emergence of azole resistance from 1994–2007 for the Radboud University Medical Center (RUMC) in Nijmegen, the Netherlands (*2*). Here, we describe trends in resistance frequency and distributions of mutations over a 23-year period, from 1994 through 2016.

All clinical *A. fumigatus* isolates cultured at RUMC are screened for azole resistance. Before 2009, isolates were screened using an agar-slant supplemented with 4 mg/L itraconazole (*2*). During 2009–2011 a 4-well plate was developed containing itraconazole (4 mg/L), voriconazole (1 mg/L), posaconazole (0.5 mg/L), and a growth control. Since 2012, we have used a commercial agar-based screening system (VIPcheck, http://www.vipcheck.nl) containing 2 mg/L of voriconazole (*3*,*4*). We performed EUCAST susceptibility testing (http://www.eucast.org) and sequencing of the *cyp51A* gene and promoter on isolates that grew on azole-containing wells (*2*).

We calculated the resistance frequency of azole-resistant *A. fumigatus* isolates for each year, using the number of cultured isolates as denominator. Furthermore, we calculated the patient-adjusted proportion of resistance for each year and for each 5-year period, using the number of patients with a resistant isolate as numerator and of culture-positive patients as denominator. We determined by χ^2^ test whether trends of resistance frequency were statistically significant (p<0.05). When 2 different resistance mechanisms were recovered from a single patient, we counted the patient once for determining the resistance proportion but used both isolates to determine the resistance frequency.

Over the 23-year period, 4,268 *A. fumigatus* isolates were cultured from 2,051 patients, a resistance frequency of 4.2% (179/4,268 isolates). Azole-resistant *A. fumigatus* was found in 109/2,051 (5.3%) culture-positive patients (Figure, panel A). The patient-adjusted resistance proportion increased from 0% in 1997 to 9.5% in 2016 (Figure, panel B). The increase of resistance was not statistically significant when the proportion of resistance for each consecutive year was analyzed. However, the 5-year proportion of resistance increased from 0.79% for 1996–2001 to 4.25% for 2002–2006, 7.17% for 2007–2011, and 7.04% for 2012–2016 (Figure, panel A). The increases in resistance for 2002–2006 compared with 1996–2001 and for 2007–2011 compared with 2002–2006 were statistically significant (p<0.05) (Figure, panel B). 

**Figure:**
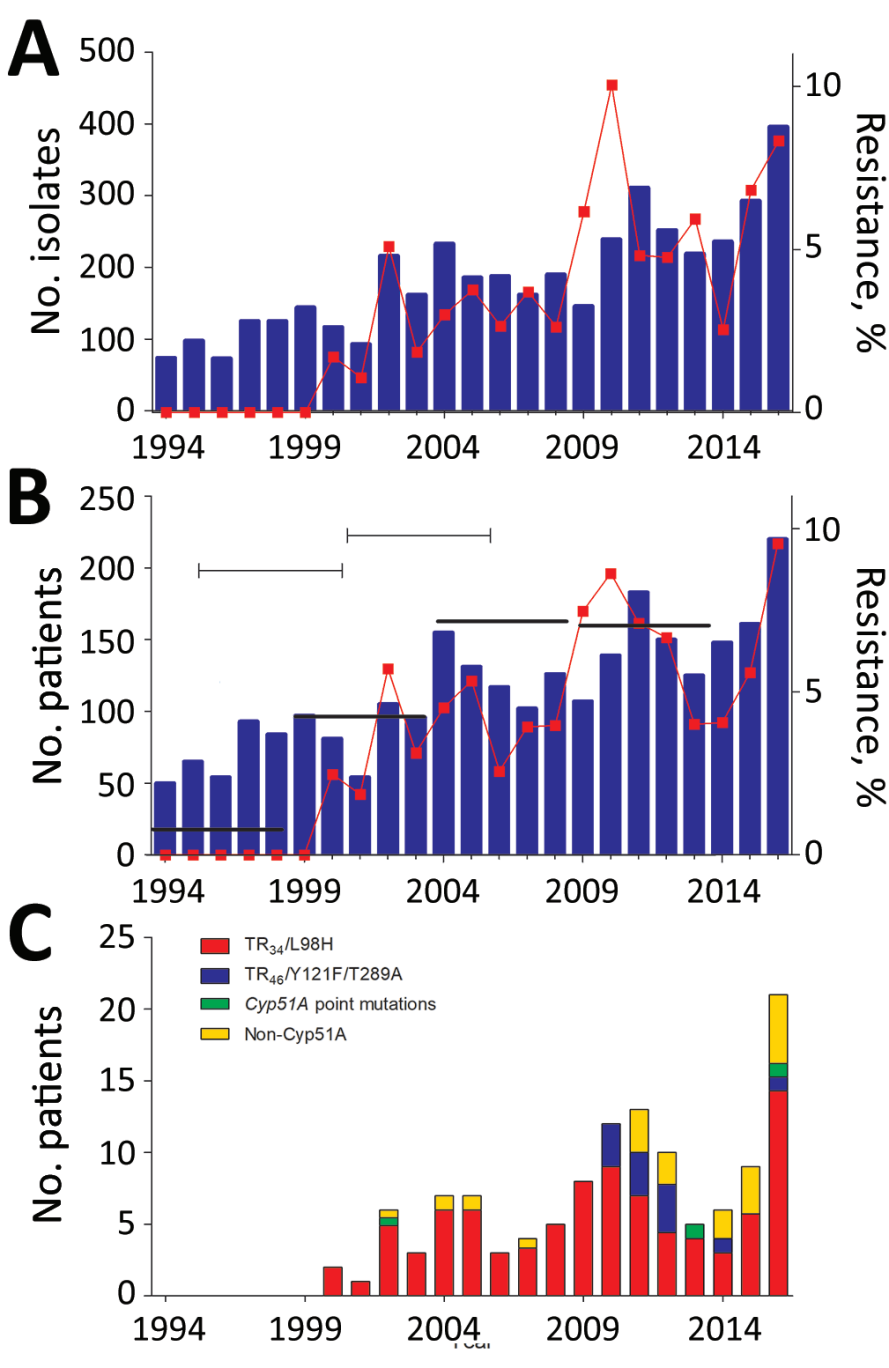
Trends in azole resistance in *Aspergillus fumigatus*, the Netherlands, 1994–2016. A) Resistance frequency by number of total cultured *A. fumigatus* isolates and percentage of azole-resistant isolates. B) Patient-adjusted proportion of resistance. Thick black horizontal bars indicate 5-year patient-adjusted proportion of resistance. p<0.05. C) Resistance mechanisms in azole-resistant *A. fumigatus.*

TR_34_/L98H was the most prevalent resistance mutation over the 23-year period; it was present in 77/109 (70.6%) patients with drug-resistant *A. fumigatus* (Figure, panel C). TR_46_/Y121F/T289A was found in 2 patients in 2010, 1 in 2011, and 1 in 2012 but only twice during 2013–2016. In recent years, resistant phenotypes without *Cyp51A* mutations were encountered more frequently than phenotypes with the mutation (Figure, panel C).

We observed an increasing trend in azole resistance prevalence in clinical *A. fumigatus* isolates until 2011, using number of patients with a positive culture as denominator. After 2011, the 5-year proportion of resistance remained stable. The advantages and disadvantages of different approaches of reporting resistance frequency remain under debate (*5*). Experts have recommended 10% resistance rate as the threshold for reconsideration of primary antifungal therapy (*6*), which indicates a need for consensus in how to determine resistance rates (*5*).

Although we found a substantial increase in azole resistance frequency over time, our study showed variation between consecutive years. Therefore, analysis of culture-positive patients over multiple years is required to determine local resistance epidemiology. Furthermore, resistance rates calculated using *Aspergillus* disease as denominator provide more information to support changes in empiric treatment decisions, but the low number of culture-positive patients in risk groups makes it difficult to obtain accurate estimates.

Various factors might have caused bias over the long period. The method of resistance detection changed from an agar-slant containing itraconazole to a system that contained 3 azoles. Voriconazole- or posaconazole-resistant isolates with low itraconazole MICs may have been initially missed, but this phenotype is very uncommon (*7*). Other factors include increased awareness of resistance, the policy to screen multiple *A. fumigatus* colonies after observation of patients with mixed azole-susceptible and azole-resistant infection (*8*), and local changes in aspergillus disease risk groups.

Resistance was dominated by environmental resistance mutations TR_34_/L98H and TR_46_/Y121F/T289A, although the number of patients with TR_46_/Y121F/T289A decreased in recent years. Furthermore, in the last 5 years, ≈15% of resistant isolates harbored a wild-type *Cyp51A* gene, suggesting that other resistance mechanisms may be emerging. Because commercial PCR tests detect only resistance mechanisms with TR_34_ (*9*) or TR_34_ and TR_46_ (*10*), our observation is relevant for using these assays in culture-negative patients.

In summary, our study indicates an increasing azole resistance trend in clinical *A. fumigatus* isolates in the Netherlands. Furthermore, our results highlight difficulties encountered in establishing local epidemiology of this resistance.
